# Characterization of Orange Peel Waste and Valorization to Obtain Reducing Sugars

**DOI:** 10.3390/molecules26051348

**Published:** 2021-03-03

**Authors:** José R. Ayala, Gisela Montero, Marcos A. Coronado, Conrado García, Mario A. Curiel-Alvarez, José A. León, Carlos A. Sagaste, Daniela G. Montes

**Affiliations:** Instituto de Ingeniería, Universidad Autónoma de Baja California, Blvd. Benito Juárez y Calle de la Normal S/N, Col. Insurgentes Este, Mexicali 21280, Baja California, Mexico; gmontero@uabc.edu.mx (G.M.); marcos.coronado@uabc.edu.mx (M.A.C.); cnrdgarciag@uabc.edu.mx (C.G.); mcuriel@uabc.edu.mx (M.A.C.-A.); jose.leon30@uabc.edu.mx (J.A.L.); carlos.sagaste@uabc.edu.mx (C.A.S.); dmontes35@uabc.edu.mx (D.G.M.)

**Keywords:** chemical composition, orange peel, proximate analysis, reducing sugars, SEM-EDS

## Abstract

Annually, millions of tons of foods are generated with the purpose to feed the growing world population. One particular eatable is orange, the production of which in 2018 was 75.54 Mt. One way to valorize the orange residue is to produce bioethanol by fermenting the reducing sugars generated from orange peel. Hence, the objective of the present work was to determine the experimental conditions to obtain the maximum yield of reducing sugars from orange peel using a diluted acid hydrolysis process. A proximate and chemical analysis of the orange peel were conducted. For the hydrolysis, two factorial designs were prepared to measure the glucose and fructose concentration with the 3,5-DNS acid method and UV-Visible spectroscopy. The factors were acid concentration, temperature and hydrolysis time. After the hydrolysis, the orange peel samples were subjected to an elemental SEM-EDS analysis. The results for the orange peel were 73.530% of moisture, 99.261% of volatiles, 0.052% of ash, 0.687% of fixed carbon, 19.801% of lignin, 69.096% of cellulose and 9.015% of hemicellulose. The highest concentration of glucose and fructose were 24.585 and 9.709 g/L, respectively. The results highlight that sugar production is increased by decreasing the acid concentration.

## 1. Introduction

**Figure 1:**
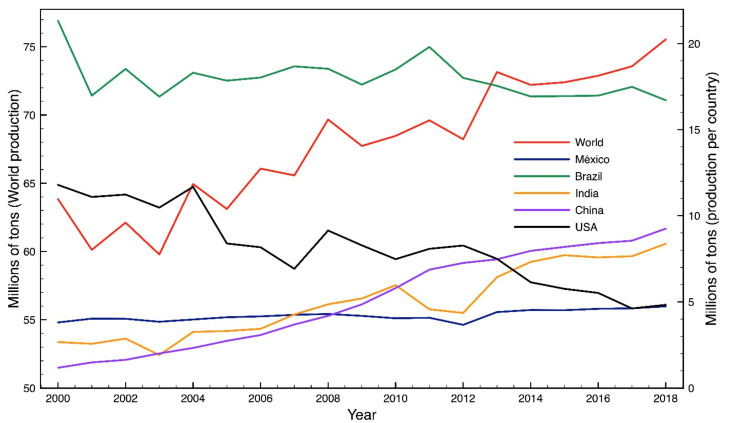
World and various countries orange production (Elaborated with data from the United Nations Food and Agriculture Organization).

In 2018, the United Nations Food and Agriculture Organization (F.A.O.) [1] estimated a world citrus production of 104.15 Mt, with 75.54 Mt corresponding to orange. During 2018, the largest orange producers worldwide were Brazil, China, India, USA and Mexico, achieving 58.10% of the total orange production. In the same year Mexico produced 4.74 Mt of orange, which represents 6.3% of the world total production [2]. Figure 1 presents the orange production of these countries compared to the world production from 2000 to 2018 [1]. In general, orange production is increasing year over year. The wastes that are generated from the orange industry include seeds, pulp, albedo and peel. Some processes take advantage of the greater amount of the fruit and used the rest in different subprocess—cattle feed, essential oil and/or limonene extraction, as well as pectin’s extraction are some of the trends applied to the orange residues. However, not all waste is used, resulting in non-hazardous waste with revalorization potential.

The orange peel is the waste with the highest volume and ease of use in the orange industry. It is estimated that around 20% of the orange is orange peel. Therefore, there is an estimation of 15.10 Mt of orange peel generation in 2018. However, before proposing a valorization route for orange peel, the physicochemical characteristics of this waste must be known. The literature indicates that the orange peel contains 23% sugar, 22% cellulose, 25% pectins and 11% hemicellulose [3,4,5]. With these values, biochemical transformations are feasible options, such as the production of bioethanol or biogas [6,7,8]. These processes require several stages of biomass treatment, so its full implementation must be studied extensively, in addition, each stage may generate a by-product [9].

In recent years, food waste valorization acquired significantly importance, whether animal or vegetable. In the case of agro-industrial waste, olive leaves have been proven to be precursors of value-added chemical compounds such as phenols [10]. Regarding organic food waste, chemical–biological treatments are used to produce hydrogen, biogas and reagents in the form of acetic acid, propionic acid and butyric acid [11]. In the case of sea biomass waste, shrimp waste is subjected to enzymatic hydrolysis to produce nitrogen compounds [12]. 

Essential oil extraction is one of the most common applications of orange peel waste. This process does not significantly transform the orange peel, it only allows the removal of the compounds that make up the essential oil such as limonene, alpha pinene, camphene, among others [13]. Normally, this type of extraction is carried out in presence of water around 100 °C. However, not taking advantage of this by-product implies two problems—the loss of a valuable material in different industries and the possibility of inhibiting microbial activity in later operations. The processes for bioethanol and biogas production include a microbial operation and different studies have shown the ability of orange essential oil to inhibit similar processes [5].

Some biomass residues that have been used as precursors of reducing sugars for bioethanol production are rice straw, rice husk, macaranga, bamboo, agave leaves, palm oil, wheat bran, sorghum stalk, sugarcane leaves and citronella residues, to mention a few [14,15,16,17,18,19,20]. To achieve the presence of reducing sugar, it is necessary to apply an integral process that can convert the hemicellulosic material of the biomass into sugars that are easily accessible to the microorganisms for fermentation. During the polymer’s degradation, important monomers such as glucose, fructose, xylose and galactose can be obtained, which by fermentation generate bioethanol. These processes can range from acid hydrolysis, enzymatic hydrolysis and simultaneous hydrolysis and fermentation [8,21,22,23,24]. The case of orange peel has been studied, where it has been reported that hydrolysis with diluted acid is one of the best options for cellulose and hemicellulose degradation [25].

Although it has been reported that diluted acid hydrolysis is the most appropriate for similar wastes, each author uses different temperature, time, and acid concentration values under 6% (*w*/*w*) [15,26]. Likewise, the scale of production of both reducing sugars and bioethanol affects the operating parameters, since high concentrations of acid deteriorate the reactors, increase the plant maintenance cost and adds costs in the form of raw materials and waste disposal [27,28]. Therefore, the objective of the present work was to determine the experimental conditions to obtain the maximum reducing sugars yield from orange peel using a diluted acid hydrolysis process once essential oil was extracted. Proximate and chemical analysis of orange peel before the hydrolysis was performed. Before and after hydrolysis, a Scanning Electron Microscopy with Energy-dispersive X-ray spectroscopy analysis (SEM-EDS) was applied to the orange peel surface.

## 2. Results

### 2.1. Results for the Proximate and Chemical Analysis of Orange Peel

Table 1 describes the proximate and chemical analysis results of the orange peel. The proximate analysis results indicate that the orange peel residue is not susceptible to its use in a thermochemical process since its primary composition is water. The volatility of various compounds in orange peel is also noticeable—the terpenes that conform the essential oils are usually volatile compounds whose phase change begins around 60 °C. In the case of solvent extractables, the orange peel shows an affinity of 40.399% for the extraction of these compounds in hot water. It is one of the reasons why the extracts prepared based on this residue are aqueous [5].

The chemical composition of the orange peel is mainly made up of cellulose, followed by lignin and then hemicellulose. Cellulose is the simplest natural polymer to convert into its monomers, which helps to improve the fermentation process [18].

### 2.2. Design of Experiments of the Dilute Acid Hydrolysis

Table 2 depicts the results obtained in the factorial designs for the concentration of glucose and fructose obtained by dilute acid hydrolysis.

The combination of factors that produces the greatest amount of glucose and fructose was 0.5% *v*/*v* of H_2_SO_4_, 125 °C and 120 min of operation. The previous combination forms up to 24.585 g/L of glucose and up to 9.709 g/L of fructose. It is important to note that at lower factors levels, it is possible to obtain a concentration of glucose and fructose above 10 g/L. At a pilot plant level, low levels of acid concentration are beneficial, since it prolongs the duration of the installations, and saves on reagent costs like H_2_SO_4_. Due to the above, the combination of factors at low levels is attractive [29]. At the maximum temperature level, sugar production increases. However, these increases do not follow the same trend if the H_2_SO_4_ concentration increases. Other biomass residues show increases in the concentration of reducing sugars while increasing the concentration of H_2_SO_4_ [29], where the orange peel shows a contrary behavior if this factor is analyzed individually.

In Minitab, two ANOVA tables were created, one with glucose concentration as a response variable and the other with fructose concentration as a response variable. The results of both analyses are shown in Table 3.

**Figure 2:**
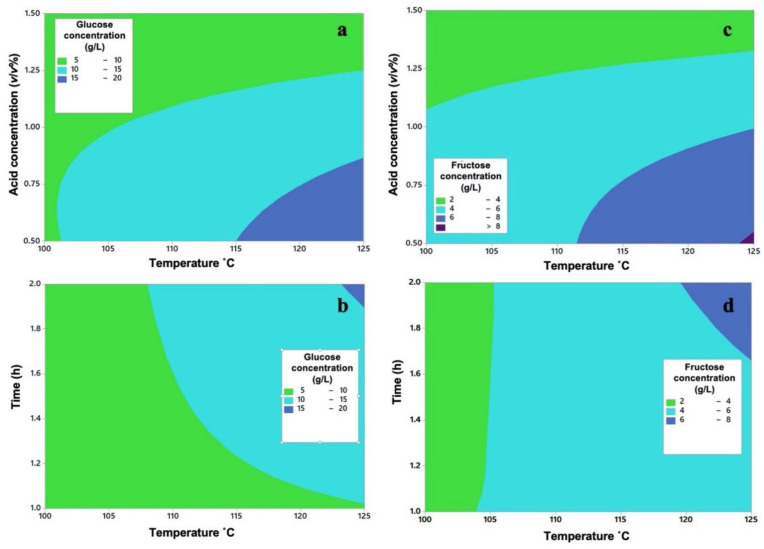
Binary interactions. (**a**) Glucose acid concentration vs. temperature; (**b**) glucose temperature vs. time; (**c**) fructose acid concentration vs temperature; and (**d**) fructose temperature vs. time.

The individual factors that turned out to be significant were the acid concentration (A) and the temperature (B) for both designs of experiments, while for glucose the operating time (C) was also an important factor. Regarding the binary interactions, the combination of acid–concentration–temperature and temperature–time was significant for both designs of experiments. Figure 2 shows the contour plots for these combinations. For the acid–concentration–temperature factors, the areas with the highest production are in the strong blue region (Figure 2a,c), which begins with a temperature of at least 112 °C and is limited to acid concentration less than 1% *v*/*v*. By increasing the temperature and decreasing the acid concentration, a clear increase in the glucose and fructose concentration can be observed. In the interaction between the temperature and the hydrolysis time (Figure 2b,d) a similar behavior is observed; the longer the time and the higher temperature, the higher the reducing sugars concentration. However, these areas are different compared with the ones in Figure 2a,c.

The contour diagram is useful because it provides factor ranges in which a response variable is similar to the ones in the design of experiments. When combining both contour diagrams for glucose and fructose, it is observed that the region with a glucose production between 15–20 g/L will occur in the ranges of 0.5–0.75% of H_2_SO_4_, 123–125 °C and 1.95–2 h. For fructose, the ranges in which a concentration between 6–8 g/L can be produced are 0.5–0.95% H_2_SO_4_, temperature of 119–125 °C and 1.7–2 h.

### 2.3. SEM-EDS Analyses

**Figure 3:**
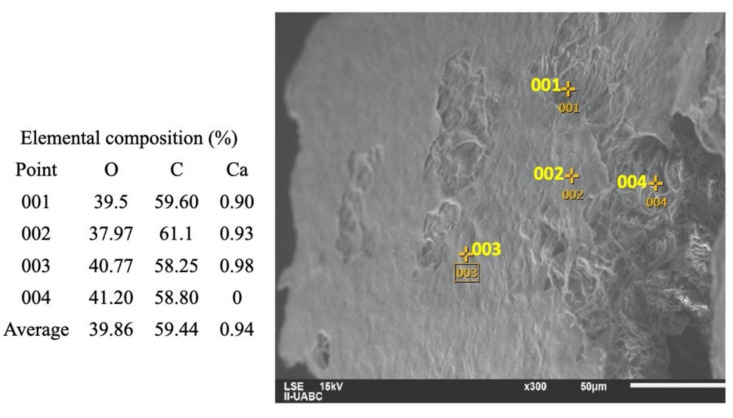
SEM-EDS analysis of orange peel before the dilute acid hydrolysis.

The image in Figure 3 shows the surface of orange peel before the addition of H_2_SO_4_. It can be seen that there is an absence of acid and NaOH. The percentage of O and C in the image was obtained without chemical modifications to the surface of the orange peel, so they are considered basal values. These values can increase or decrease depending on the type of reaction that occurs with H_2_SO_4_ and NaOH.

**Figure 4:**
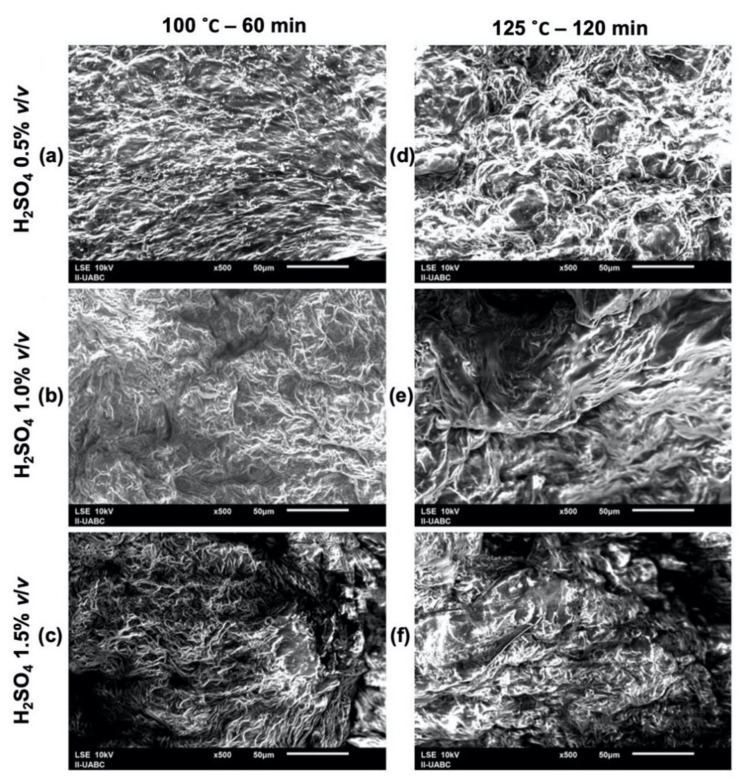
Surface comparison of the hydrolyzed orange peel samples, (**a**) H_2_SO_4_ 0.5% *v*/*v*, 100 °C and 60 min, (**b**) H_2_SO_4_ 1.0% *v*/*v*, 100 °C and 60 min, (**c**) H_2_SO_4_ 1.5% *v*/*v*, 100 °C and 60 min, (**d**) H_2_SO_4_ 0.5% *v*/*v*, 125 °C and 120 min, (**e**) H_2_SO_4_ 1.0% *v*/*v*, 125 °C and 120 min, (**f**) H_2_SO_4_ 1.5% *v*/*v*, 125 °C and 120 min.

The images presented in Figure 4 were taken based on the results of the design of experiments. For this, the combinations of temperature–time at low level and high level were chosen. It can be seen that the surfaces deteriorate due to the hydrolysis, which increases from top to bottom and from left to right. As the H_2_SO_4_ concentration increases in the hydrolysis, the surface contracts and becomes irregular.

**Figure 5:**
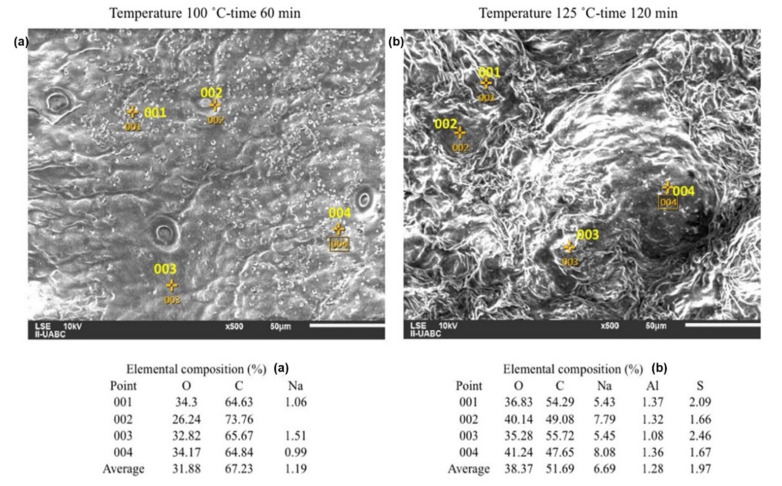
EDS analyses for orange peel treated with H_2_SO_4_ at 0.5 % *v*/*v*. (**a**) for 100 °C and 60 min. (**b**) for 125 °C and 120 min.

The operation conditions for Figure 5a were 60 min and 100 °C, and for Figure 5b were 120 min and 125 °C. The elemental analysis indicates that S was not enough to be deposited on the surface of Figure 5a. In the case of Figure 5b, an average of 1.97% of S appears on the surface. Because the neutralizations were carried out with NaOH, the presence of Na is remarkable in both cases, being 5.6 times higher for Figure 5b. The results for Figure 5b are capable of producing on average 21.887 g/L of glucose and 9.286 g/L of fructose. If these values are compared with those in Figure 5a, which were 10.659 g/L of glucose and 4.872 g/L of fructose, the decrease of C in Figure 5b can be explained, since when the H_2_SO_4_ reacts under these conditions it generated a greater quantity of reducing sugars, as a result of a greater consumption of C. In some cases, the equipment detects Al in the samples, which is attributed to parts of the orange peel that had direct contact with the container where they were placed. With respect to the surface, Figure 5a is more uniform compared to Figure 5b. In addition, in the latter, rougher areas can be seen.

**Figure 6:**
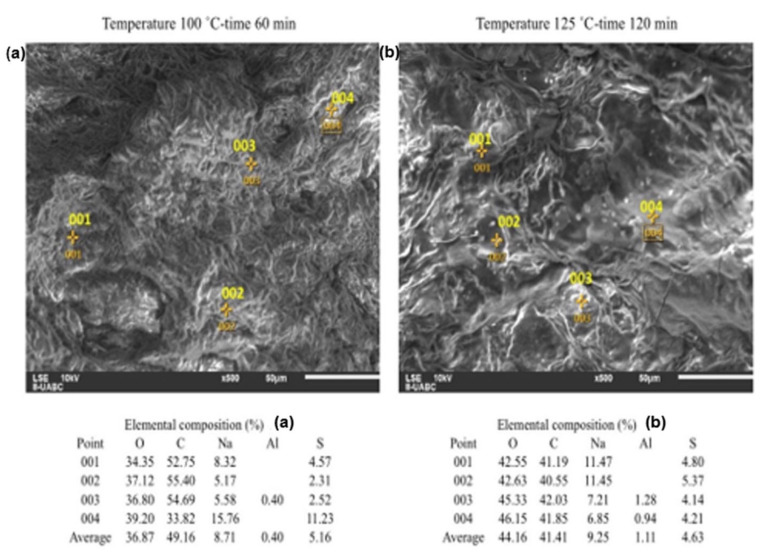
EDS analyses for orange peel treated with H_2_SO_4_ at 1.0 % *v*/*v*. (**a**) for 100 °C and 60 min. (**b**) for 125 °C and 120 min.

The operating conditions of the sample in Figure 6a were 60 min and 120 °C, and for Figure 6b, the conditions were 120 min and 125 °C. For both cases of Figure 6a, the concentration of S at the surface is appreciable, with a similar average at the points shown. There is an average concentration below 1.20% of Al in the two figures, which is attributed to the lid of the container where the hydrolysate reaction occurs. The concentration of O increases from Figure 6a to Figure 6b—since both NaOH and H_2_SO_4_ contain O, there is an addition of these materials to the surface of the orange peel. In the case of C, a decrease occurs in Figure 6b, which is attributed to its consumption for the generation of glucose and fructose. On average, Figure 6a generates 8.105 g/L of glucose and 3.708 g/L fructose, in contrast to the 16.277 g/L of glucose and 7.356 g/L of fructose that are generated in Figure 6b. The generation of both sugars in Figure 6b is doubled compared to Figure 6a, even though the elemental compositions of S and Na remain similar. Visually, both samples present surfaces worn by hydrolysis.

**Figure 7:**
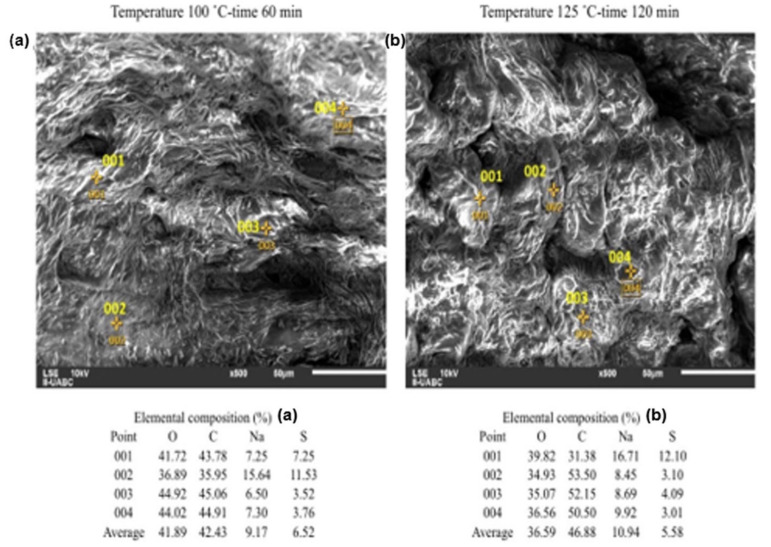
EDS analyses for orange peel treated with H_2_SO_4_ at 1.5 % *v*/*v*. (**a**) for 100 °C and 60 min. (**b**) for 125 °C and 120 min.

The operating conditions of Figure 7a were 60 min and 100 °C and for Figure 7b 120 min and 125 °C. For the two cases the concentration of H_2_SO_4_ used was 1.5% *v*/*v*. For this EDS, it is important highlight that the average glucose and fructose productions in both hydrolysis reactions were similar at 6.560 g/L of glucose and 3.129 g/L of fructose for Figure 7a, while for Figure 7b it was 8.657 g/L of glucose and 3.652 g/L of fructose. With the above, a similar behavior was expected in the element’s percentage compositions, an effect that occurs for both Na and S. In addition, C now has a higher percentage composition in Figure 7b than in Figure 7a. The operating conditions of the sample in Figure 7b indicate that the hydrolysis is not as satisfactory as in the other EDS. Visually, Figure 7b shows a lower surface uniformity if compared to its counterpart Figure 7a.

## 3. Discussion

According to proximate analysis results, thermochemical applications such as direct combustion of orange peel are not recommended due to the high moisture content. Biological applications must be carefully studied, since the amount of volatile compounds such as limonene will prevent the growth of certain microorganisms. The presence of volatile compounds is evident. Regardless of the revalorization procedure for orange peel, these compounds that comprise orange peel essential oil must be considered. As an industrial process that seeks the use of orange peel for the production of reducing sugars and bioethanol, it must consider the essential oil as a by-product to help the profitability of the process. The results of orange peel chemical composition are linked to the quantification of glucose and fructose, since cellulose degrades into these monomers when is hydrolyzed.

Regarding the results from the design of experiments, the conditions under which the greatest amount of reducing sugars in the form of glucose and fructose are produced were—H_2_SO_4_ concentration 0.5% *v*/*v*, temperature of 125 °C and hydrolysis time 120 min. The above combination gives an average glucose and fructose concentration of 21.887 and 9.286 g/L, respectively. When increasing the H_2_SO_4_ concentration, a decrease in the sugars produced was observed. Presenting on average, at the highest levels of the factors, a production of 8.657 g/L of glucose and 3.652 g/L of fructose, up to three times lower compared to the conditions of maximum production. The glucose and fructose concentrations achieved according to the design of experiments may vary even under strict operating conditions. This is due to the fact that orange peel waste can have a different composition according to the geographical position, soil type where orange was grown and weather conditions. However, working with orange peel waste, the tendency set by the parameters of the design of experiments must be maintained, at low acid concentration, 125 °C for 2 h.

The results of the SEM-EDS analysis yield morphological and elemental information of the surface that confirm the information obtained by the design of experiments. An important aspect to highlight is that, unlike other biomass materials such as corn, the concentration must remain at low levels, which is favorable.

Both the percentage of O and Na in the sample increase with increasing H_2_SO_4_ concentration, due to the greater need for NaOH to maintain the pH of the hydrolysate between 4.8 and 5.2. Likewise, if the concentration of H_2_SO_4_ increases, the % of C decreases in the mixture, which reacts to form the sugars dissolved in the hydrolysate. The previous behavior is not fulfilled in the hydrolysate with the maximum factor levels, since the temperature–time interaction causes a low reaction yield in the generation of reducing sugars, and that the SEM-EDS detects a % of C that was lower that its counterparts.

## 4. Materials and Methods

### 4.1. Proximate and Chemical Analysis of Orange Peel

For the orange peel proximate analysis, 1 g of freshly peeled sample was weighed, which included both albedo and peel and placed inside a crucible of known mass. The crucible and the sample were placed in a muffle at 45 °C for 48 h [30]. Once it reached the desired time in the muffle, the sample was cooled to room temperature in a desiccator and the mass of the crucible was measured. The moisture percentage was calculated by weight difference. To calculate the volatiles percentage, the moisture-free sample was brought to a temperature of 950 °C in the absence of air for 7 min, then it was cooled on a ceramic surface in a desiccator at room temperature and the mass of the crucible was measured. The difference in mass was calculated as the volatile percentage [31]. For the ash analysis, the sample resulting from the determination of volatiles was subjected to 580 °C for 4 h, cooled in a desiccator with ceramic surface and the mass of the crucible was measured, the difference in percentage was recorded as ash content [32]. Finally, the remaining residue in the crucible was quantified and considered as the fixed carbon percentage. The moisture, volatiles, ash and fixed carbon analyses were carried out in triplicate.

The chemical analysis of the orange peel began by measuring 4 g of moisture-free sample and then placing it in a thimble of known mass. Subsequently, the thimble was placed inside soxhlet extraction equipment with 200 mL of acetone as solvent and boiled for 8 h [33]. At the end of the extraction, the thimble was dried in a muffle at 105 °C for 3 h, cooled to room temperature in a desiccator and then the mass of the thimble with sample was measured. The acetone extractables percentage was calculated by weight difference.

For the determination of hot water extractables, 3 g of moisture-free sample of orange peel and free from acetone extractables was used. The sample was placed in a 250 mL ball flask, 200 mL of hot water was added and it was boiled in a water bath with condensation refluxed for 3 h [34]. At the end of the extraction, a filter paper was weighed and used in a büchner funnel to separate the sample from the hot water. The filter paper was brought to 105 °C for 4 h in a muffle, dried at room temperature in a desiccator and the mass of the filter paper with the orange peel was measured. The hot water extractables percentage was determined by weight difference. The resulting orange peel was considered free of total extractables.

The lignin content determination was carried out using 1 g of orange peel free of moisture and free of total extractables, which was mixed with 15 mL of H_2_SO_4_ at 72%, stirred for 1 min at 400 rpm and for 2 h at 200 rpm. Then, the solution was placed in a 1 L Erlenmeyer flask with 560 mL of distilled water and it was boiled with condensation reflux for 4 h [35]. To filter the sample, a filter paper was weighed and used with a bücher funnel. The filter paper was heated to 105 °C and 4 h, then cooled to room temperature in a desiccator and the mass of the paper was measured. The lignin percentage was calculated by weight difference.

For the holocellulose content, 2 g of moisture-free orange peel that was free of total extractables was added inside an Erlenmeyer flask. Also, 150 mL of distilled water, 0.2 mL of acetic acid and 1 g of sodium chlorite were used to complete the mixture [36]. The flask was sealed and placed in a water bath at 75 °C for 5 h. Every hour, 0.2 mL of acetic acid and 1 g of sodium chlorite were added, until reaching a total of 1 mL of acetic acid and 5 g of sodium chlorite. The procedure ended at the fifth hour and the sample was removed from the water bath. A filter paper with known mass was used in a büchner funnel to filter the sample. During the filtration, 500 mL of distilled water was used as a wash until the yellow coloration of the sample was removed. The content of the paper was brought to 105 °C for 4 h, then it was cooled in a desiccator to finally measure its mass on a balance. The holocellulose content was measured by weight difference.

The cellulose content determination used 2 g of sample resulting from the holocellulose determination, which was added to an Erlenmeyer flask with 10 mL of 17.5% NaOH, and allowed to rest for 5 min at 20 °C [37]. A total of 5 mL of 17.5% NaOH was added every 5 min until reaching 25 mL of NaOH. Then, the last 5 mL of NaOH the sample was allowed to rest for 30 min. Then, 33 mL of distilled water was added and the mixture was left to rest for 60 min. The content of the flask was neutralized with 15 mL of acetic acid and NaOH at 8.3% until pH of 7 was reached, then it was filtered using a filter paper of known mass. After the filtration, the filter paper and its content were dried at 105 °C for 4 h and allowed to cool in a desiccator. Finally, the filter paper was weighed and the cellulose content was measured by weight difference. The hemicellulose content was determined by percentage difference between the holocellulose and cellulose content. 

### 4.2. Design of Experiments for the Orange Peel Diluted Acid Hydrolysis

A design of experiments of the factorial design type applied to the diluted acid hydrolysis of orange peel was developed to contrast the obtained reduced sugars [38,39]. The purpose of a design of experiment is to identify the variables that significantly affect a process from a statistical point of view, adjusting the operation parameters to achieve the best result according to the desired application [26].

The selected factors were—(A) H_2_SO_4_ concentration, (B) temperature, (C) hydrolysis time. A total of 3 levels were specified for factor (A) and 2 levels for factors (B) and (C), with 2 replicates per combination of variables. The amount of hydrolysis performed for this design of experiment was 24 experiments. The levels for the factors were concentration of H_2_SO_4_ 0.5%, 1.0% and 1.5% all in volume percent, for the temperature 100 °C and 125 °C and for the hydrolysis time 1 and 2 h. The purpose of the factorial design is to identify which individual factors and combinations thereof are capable of significantly affecting the production of sugars. The results of the sugar production were analyzed in Minitab^®^ where the important process variables were identified.

Orange peel needs a pretreatment before the hydrolysis, a series of orange peel hydrodistillations were carried out to remove the essential oil. The essential oil was stored in amber vials and refrigerated at 4 °C [13]. The orange peel without essential oil was heated 24 h at 65 °C [22,40,41]. The previous procedure was repeated, until 100 g of dried orange peel was obtained, then its size was reduced in a mortar and placed in a desiccator, until later use.

A furnace was preheated for 30 min to a temperature set in the design of experiments. At the same time, 2 g of dried orange peel was placed in a 100 mL flask, adding 60 mL of the H_2_SO_4_ solution (1:30 ratio). The flask was placed in the furnace by the time of hydrolysis, and once finished, it was removed and cooled to a temperature of 30 °C. The acid pH of the hydrolysate was brought to 4.8–5.2 with NaOH 0.5 N, then it was filtered and stored at 4 °C [42]. The filtered orange peel was dried at 65 °C for 24 h and placed in a desiccator for further analysis by SEM.

For the reducing sugars identification, the 3,5-DNS method was chosen [43,44]. This method has proved its validation for reducing sugars identification by using known standard solutions [45,46,47]. Two calibration curves were prepared, one with glucose and the second with fructose. In both curves, 10 points were used, whose concentration ranges ranged from 0.2 to 2.0 g/L. Once the calibration curve was ready, each hydrolysate sample was diluted 10 times and 3 mL of it was taken. Subsequently, 3 mL of the prepared DNS reagent was added to each dilution, to a total of 6 mL, in a 1:1 ratio (sample—DNS). Each of the preparations was heated for 5 min at 100 °C, to generate the chemical reaction that gives the color to the sample and then cooled to 25 °C. Also, a DNS solution blank was prepared. The quantification of reducing sugars was achieved by a Perkin Elmer Lambda 25 UV-Vis Spectrometer (Waltham, MA, USA), and the method worked at 575 nm. All the hydrolysate samples treated were contrasted with the 2 calibration curves and their readings were recorded.

### 4.3. SEM-EDS

The samples for SEM were placed on a conductive plate with carbon tape without metallic coating. A total of 6 samples were taken from 24 of the design of experiments, which had the highest amount of sugar produced. The equipment used was a JEOL JSM-6010LA analytical scanning electron microscope (Tokyo, Japan), with a working distance of 11 mm, acceleration voltage of 10 kV and under vacuum of 50 Pa [48].

The general procedure of this work can be seen in Table 4. This scheme includes the analysis of reducing sugars and the SEM-EDS of the samples after hydrolysate.

## 5. Conclusions

Under the selected parameters of the design of experiments, the maximum concentration of glucose and fructose was achieved with an acid concentration of 0.5%, at 125 °C for 2 h. However, this does not mean that they are the best conditions to operate a hydrolysis process applied to orange peel waste. Future research could study the effect of reducing the concentration of H_2_SO_4_ to find the turning point, where the reducing sugars concentration starts to decrease. In addition, the effect of the volume ratio of H_2_SO_4_ and orange peel waste can be addressed, since in all the experiments it remained constant. While this methodology allows essential oil extraction from orange peel, other value-added products such as pectins can be explored before this waste is subjected to fermentation processes to obtain alcohols.

Although it is possible to modify the pretreatment of the orange peel to improve the production of sugars, the revaluation of a waste must be an integral process, which follows the principles of the biorefinery concept. That is, not just looking for punctual application for the given waste, but obtaining multiple products from the same source in such a way that can be scalable to a commercial level. Depending on the desired production scale, either at the laboratory or pilot plant level, this work provides the concentration of reducing sugars in the form of glucose and fructose, according to the 12 combinations proposed in the design of experiments.

## Figures and Tables

**Table 1:** Proximate and chemical analysis of orange peel.

Analysis		Composition (% in Weight)	Standard Deviation
Proximate	Moisture	73.530%	0.477%
	Volatiles	99.261%	0.074%
	Ash	0.052%	0.004%
	Fixed carbon	0.687%	0.078%
Chemical	Acetone extractables	6.821%	0.604%
	Hot water extractables	40.399%	2.595%
	Lignin determination	19.801%	3.595%
	Holocellulose determination	78.110%	4.404%
	Cellulose determination	69.096%	9.015%
	Hemicellulose determination	5.433%	5.433%

**Table 2:** Glucose and fructose concentration results for the factorial design.

Run	H_2_SO_4_ Concentration (%*v*/*v*) (A)	Temperature (°C) (B)	Time (h) (C)	Glucose (g/L)	Average Glucose (g/L)	Standard Deviation (Glucose)	Fructose (g/L)	Average Fructose (g/L)	Standard Deviation (Fructose)
1,2	0.5	100	1	11.302 10.016	10.659	0.909	4.651 5.092	4.871	0.312
3,4	0.5	100	2	7.940 8.821	8.380	0.623	3.367 3.499	3.433	0.093
5,6	0.5	125	1	13.843 17.009	15.426	2.239	5.242 8.936	7.089	2.612
7,8	0.5	125	2	24.585 19.189	21.887	3.816	9.709 8.862	9.285	0.599
9,10	1	100	1	8.212 7.998	8.105	0.151	4.231 3.184	3.707	0.740
11,12	1	100	2	9.500 10.309	9.904	0.572	3.580 5.600	4.59	1.428
13,14	1	125	1	9.686 11.373	10.529	1.193	4.049 5.090	4.569	0.736
15,16	1	125	2	16.727 15.826	16.276	0.637	6.803 7.908	7.355	0.781
17,18	1.5	100	1	7.080 6.039	6.559	0.736	3.880 2.377	3.128	1.063
19,20	1.5	100	2	3.397 4.192	3.794	0.562	1.768 1.742	1.755	0.018
21,22	1.5	125	1	3.064 4.274	3.669	0.856	1.829 2.015	1.922	0.132
23,24	1.5	125	2	8.604 8.709	8.656	0.074	3.600 3.704	3.652	0.074

**Table 3:** ANOVA data for glucose and fructose.

Variation Source	Glucose					Fructose				
	Square Sum	Freedom Degrees	Mean Square	F_0_	F Critic	Square Sum	Freedom Degrees	Mean Square	F_0_	F
										Critic
Acid concentration (A)	292.829	2	146.415	71.54	3.89	52.911	2	26.456	26.06	3.89
Temperature (B)	140.568	1	140.568	68.68	4.75	25.911	1	25.577	25.20	4.75
Time (C)	32.441	1	32.441	15.85	4.75	3.813	1	3.813	3.76	4.75
AB	67.025	2	33.512	16.37	3.89	13.803	2	6.902	6.80	3.89
AC	7.251	2	3.625	1.77	3.89	3.267	2	1.634	1.61	3.89
BC	69.629	1	69.629	34.02	4.75	12.447	1	12.448	12.26	4.75
ABC	6.403	2	3.201	1.56	3.89	0.787	2	0.393	0.39	3.89
Error	24.560	12	2.047			12.181	12	1.015		
Total	640.705	23				124.786	23			

**Table 4:** Procedure of the presented work.

Sample	Stage	Analysis	Conditions and Equipment	Reference
Orange peel	Proximate analysis	Moisture	45 °C for 48 h inside a muffle	[30]
		Volatiles	950 °C for 7 min without air	[31]
		Ash	580 °C for 4 h inside a muffle	[32]
		Fixed carbon	The difference in % of the sample, moisture, volatiles and ash analysis	[32]
	Chemical analysis	Acetone extractable	8 h Soxhlet extraction with acetone	[33]
		Water extractable	3 h boiling water with condenser reflux	[34]
		Lignin %	4 h, 15 mL of H_2_SO_4_, stirring and 560 mL of distilled water	[35]
		Holocellulose %	150 mL water, 0.2 mL of acetic acid and 1 g of sodium chlorite per hour for 4 h.	[36]
		Cellulose %	25 mL of NaOH at 17.5 %, 100 mL of NaOH at 8.3%, 10 mL of acetic acid and water for 105 min.	[37]
	Essential oil extraction	Hydrodistillation	65 g of orange peel, 90 min, orange peel grinding of 1 min, 500 mL of water	[13]
	Hydrolysis	Diluted acid hydrolysis	H_2_SO_4_ concentration, time and temperature according to the factorial design	[38,39]
		pH stabilization	NaOH at 0.5 N until a pH of 4.8–5.2 was reached	[42]
		Orange peel drying	65 °C for 24 h	
	SEM-EDS	SEM micrography	JEOL JSM-6010LA SEM, working distance 11 mm, 10 kV, 50 Pa, 300x, 500x	[48]
		EDS analysis		
Hydrosylate	Reducing sugars determination	3,5 DNS reagent	2.5 g of 3,5-Dinitric salicylic acid, 7.5 g of mixed potassium sodium tartrate, 4 g of NaOH in 250 mL of water	[44,45,47]
		Calibration curve	Glucose and fructose calibration curves prepared from 0.2 g/L to 2 g/L with water	[43,44]
		Sample preparation	1:10 dilution of the hydrosylate in distillated water and 1:1 diluted hydrosylate with DNS reagent	
		Reducing sugars quantification	Perkin-Elmer Lambda 25 UV-Vis Spectrometer at 575 nm measured in quartz cell	[43,44,47]

## Data Availability

The data that support the findings of this study are available from the corresponding authors upon reasonable request.

## Sample Availability

Not available.
